# Clustered Routing Using Chaotic Genetic Algorithm with Grey Wolf Optimization to Enhance Energy Efficiency in Sensor Networks

**DOI:** 10.3390/s24134406

**Published:** 2024-07-07

**Authors:** Halimjon Khujamatov, Mohaideen Pitchai, Alibek Shamsiev, Abdinabi Mukhamadiyev, Jinsoo Cho

**Affiliations:** 1 Department of Computer Engineering, Gachon University, Seognam-daero, Sujeong-gu, Seongnam-si 1342, Gyeonggi-do, Republic of Korea; kh.khujamatov@gmail.com; 2 Department of Computer Science and Engineering, National Engineering College, Kovilpatti 627011, Tamilnadu, India; kmpcse@nec.edu.in; 3 Department of Data Communication Networks and Systems, Tashkent University of Information Technologies Named after Muhammad al-Khwarizmi, Tashkent 100200, Uzbekistan; a.shamsiyev@tuit.uz

**Keywords:** chaotic genetic algorithm, clustering, energy efficiency, grey wolf optimizer, routing, sensor networks

## Abstract

As an alternative to flat architectures, clustering architectures are designed to minimize the total energy consumption of sensor networks. Nonetheless, sensor nodes experience increased energy consumption during data transmission, leading to a rapid depletion of energy levels as data are routed towards the base station. Although numerous strategies have been developed to address these challenges and enhance the energy efficiency of networks, the formulation of a clustering-based routing algorithm that achieves both high energy efficiency and increased packet transmission rate for large-scale sensor networks remains an NP-hard problem. Accordingly, the proposed work formulated an energy-efficient clustering mechanism using a chaotic genetic algorithm, and subsequently developed an energy-saving routing system using a bio-inspired grey wolf optimizer algorithm. The proposed chaotic genetic algorithm–grey wolf optimization (CGA-GWO) method is designed to minimize overall energy consumption by selecting energy-aware cluster heads and creating an optimal routing path to reach the base station. The simulation results demonstrate the enhanced functionality of the proposed system when associated with three more relevant systems, considering metrics such as the number of live nodes, average remaining energy level, packet delivery ratio, and overhead associated with cluster formation and routing.

## 1. Introduction

Wireless sensor networks (WSNs) are networks composed of several nodes strategically deployed in the environment, where sensors are tasked with sensing and transmitting information to the sinks and base stations. However, these sensors have limited battery power and are very difficult to recharge following deployment [1]. Therefore, the present study’s objective was to reduce the energy exhaustion of sensors and thereby extend the overall lifetime of the sensor network. To minimize network energy consumption, nodes are grouped into clusters [2], with each cluster containing a designated cluster head collecting and aggregating information from its members. Subsequently, the cluster head forwards the collected information to the sink using either a single- or multi-hop approach. The selection of cluster heads is a challenging task, and various techniques have been employed to achieve an optimal selection of cluster heads [3,4].

Because the direct transmittance of data to the base station demands higher energy expenditure from the cluster heads, clustered sensor networks require a routing protocol that prioritizes minimizing energy consumption while selecting the optimal path from the clusters to the base station [5]. However, the construction of a clustering-based routing algorithm with high energy efficiency and increased packet transmission rate for large-scale sensor networks is an NP-hard problem [6]. In other words, the process of selecting optimal cluster heads and establishing efficient routing paths for large-scale sensor networks entails high time complexity. This issue can be efficiently solved by metaheuristic algorithms [7,8] and swarm intelligence [9,10].

This paper introduces an energy-saving clustering mechanism utilizing a chaotic genetic algorithm (CGA) coupled with the construction of an energy-efficient routing system using swarm intelligent grey wolf optimization (GWO). The proposed system, named ‘chaotic genetic algorithm–grey wolf optimization (CGA-GWO)’ is designed to minimize overall energy consumption by selecting energy-aware cluster heads and devising an optimal routing path to reach the base station. The key contributions and novelty of this study are summarized as follows:

- Chaotic systems, including logistic and tent maps, are employed to generate the initial population and govern the crossover and mutation processes of the genetic algorithm for the selection of cluster heads. The fitness function is specifically designed to identify chromosomes with higher residual energy and designate them as cluster heads. To accelerate genetic algorithm convergence, optimal cluster heads are selected by the elitist selection method in place of the roulette selection method.
- To determine an energy-efficient routing path to the base station, the GWO method was selected for its ability to yield optimal solutions across multiple iterations. The efficacy of these solutions was evaluated using a fitness function, where a higher fitness value signifies a reduction in overall distance, fewer hops, and minimized energy consumption along the routing path.

The remainder of this paper is organized as follows: Section 2 presents an investigation of pertinent mechanisms concerning energy-efficient cluster-based routing, along with essential background information on CGA and GWO; Section 3 provides a detailed explanation of the proposed CGA-GWO algorithm; Section 4 delves into a discussion of simulation results; finally, Section 5 concludes the paper.

## 2. Related Work

This section outlines recent developments in the study of energy-aware cluster routing within sensor networks. In recent years, a trend has emerged towards incorporating hybrid approaches, including swarm-based metaheuristic optimization, aimed at lowering energy depletion and thereby extending network lifetime. Ajmi et al. [11] proposed an energy-saving clustering approach known as the chicken-swarm-based genetic algorithm, which incorporates chicken-swarm-based optimization, cluster head selection, multi-weight clustering, a genetic algorithm, and cluster communication. Although this approach is notably effective in terms of energy efficacy, end-to-end latency, ratio of delivered packets, and network throughput, it might not be suitable for large-scale applications due to the possibility of communication delays.

Jia et al. [12] proposed a clustering algorithm that utilizes ant colony optimization through adaptive chaos. To disrupt the pheromone along the route, the algorithm employs chaotic mapping while enhancing the transition probability using an adaptive technique. Using this approach, the local pheromone is updated and ants are sent out to adjust the present pheromone levels using a chaos factor. Nonetheless, this algorithm does not distribute transmission tasks effectively in scenarios involving a relationship between the cluster head and sink node. Wang et al. [13] presented a cluster routing protocol that incorporates a self-organizing map neural network and firefly algorithm. Using the ant colony optimization algorithm, intercluster routing is used to select the next hop node by considering factors such as energy, distance, and node angle. The updating process of pheromones relies on the geometric coefficient of variation, and the routing path is improved by linking energy and distance.

Majeed et al. [14] introduced a fuzzy-based genetic algorithm designed for cluster formation and cluster head selection, achieving fuzzy rule optimization and output value modification for membership in fuzzy logic. In addition, the ant colony process was employed to estimate the shortest path from each cluster head to the base node. A limitation of this approach lies in its focus on short routing, which leads to a suboptimal load balance in the network. Ram et al. [15] introduced an effective approach for selecting cluster heads using the K-genetic algorithm. In this method, sensors are assembled into clusters based on their locations using k-means clustering, and a genetic algorithm is implemented to determine the most suitable cluster head. A trust-based firefly algorithm is applied to ensure secure and optimal routing.

Agrawal [16] proposed a method for selecting cluster heads through the application of grey wolf swarm intelligence. The conventional GWO algorithm was tailored to the specific objective of selecting cluster heads in sensor networks, where the cluster formation function is determined by various factors including the balancing feature of the cluster heads, residual energy, average distance of each cluster, and sink distance. Joseph et al. [17] proposed an enhanced chaotic GWO algorithm to enhance energy awareness in sensor networks, focusing on the selection of optimal cluster heads and routing paths. This approach aims to increase the percentage of packets delivered inside the sensor network while reducing node energy consumption.

Patra et al. [18] introduced an energy-efficient clustering technique that employs a genetic algorithm. For shortest-path routing, they utilized ad hoc multipath routing by adapting grey wolf intelligence. Their approach identifies an optimal multipath route from paths generated during on-demand routing, where gray wolf intelligence is used to anticipate the optimal path. Singh et al. [19] introduced an energy-saving routing algorithm that employs fuzzy GWO, aiming to minimize power consumption and achieve a balance in power usage among nodes in the sensor network. Additionally, the algorithm facilitates the reliable collection of link data by designated nodes and enhances throughput by mitigating traffic resulting from buffer occupancy in the nodes.

Gunjan et al. [20] proposed a protocol based on a metaheuristic approach for clustering and routing in sensor networks, where a heuristic search is deployed to select cluster heads by considering three distinct fitness factors: the residual energy of each cluster head, the distance between each cluster head and the sink, and intercluster isolation. To direct data towards the base station, three additional fitness functions are applied targeting the residual energy of the next hops, distance to the next hop node from each cluster head, and total hop count. Liu et al. [21] introduced the LEACH protocol based on a genetic algorithm comprising three phases for each round: preparation, setup, and data transmission. During the preparation phase, all nodes transmit messages indicating their candidacy statuses as cluster heads and share their locations with the base station. Using a genetic algorithm, the base station determines the optimal probability of each node becoming a cluster head. Subsequently, in the setup phase, the base station broadcasts a message containing the probability of all nodes forming clusters. The LEACH protocol was adopted for the setup and data transmission phases of each round of the protocol.

In all aforementioned studies, the genetic algorithm was used to encode clustering and routing processes within a single chromosome, resulting in the global estimation of energy. In addition, previous hops were not considered as parameters in the fitness functions for network load balancing. At present, there is a notable focus on leveraging the combined power of the chaos genetic algorithm (CGA) and grey wolf optimization (GWO) to enhance energy efficiency in sensor networks. This combination is gaining traction as a promising strategy to optimize energy consumption, extend network lifetime, and improve overall performance in sensor network deployments. The chaos genetic algorithm introduces chaotic dynamics into the genetic algorithm framework, promoting exploration and preventing premature convergence. This chaotic exploration enables CGA to effectively search for optimal solutions in complex and dynamic environments, making it well suited for addressing the challenges inherent in energy optimization in sensor networks.

On the other hand, grey wolf optimization mimics the social hierarchy and hunting behavior of grey wolves to optimize solutions in a distributed manner. GWO’s hierarchical search strategy allows for efficient exploration of the solution space, enabling the identification of energy-efficient configurations and resource allocation strategies within sensor networks. The synergy between CGA and GWO has led to significant strides in enhancing energy efficiency within sensor networks. This progress is evident across several avenues, notably through the development of energy-aware clustering mechanisms, routing protocols, and energy-harvesting optimization techniques. In contrast, the presently proposed CGA-GWO algorithm encodes clustering and routing in different chromosomes while using energy consumption and previous hops as parameters to evaluate the fitness function.

### 2.1. Chaotic Genetic Algorithm

The genetic algorithm, much like other evolutionary algorithms, is founded upon randomness. Consequently, local convergence and high tolerance for results owing to randomness represent primary drawbacks of the genetic algorithm [22]. To enhance the performance of the conventional genetic algorithm, chaotic maps are employed in place of conventional random functions to generate random values. As a nonlinear phenomenon in nature, chaos can be used to mirror system complexity. Furthermore, the role of randomness introduced by chaotic dynamics resembles that of random variables. Optimization methods leverage chaotic systems to generate data with random values [23].

Chaotic systems, such as Hennon and logistic maps, can substitute for randomness in the initial population, as well as the mutation, and crossover processes of the genetic algorithm. As the initial population generates random solutions, the randomness in the crossover creates new offspring. The randomness inherent to the mutations alters some genes in the offspring [24]. Thus, the significant attributes of chaotic systems—namely pseudo-randomness and determinism—serve as compelling reasons for employing them in place of random processes within the chaotic genetic algorithm. This choice helps circumvent local convergence issues, thereby enhancing the traditional genetic algorithm’s performance [25].

### 2.2. Grey Wolf Optimization

Mirjalili et al. [26] introduced the GWO algorithm inspired by the stalking behavior of grey wolves in nature. Specifically, gray wolves can be classified as alpha, beta, delta, or omega. The GWO algorithm involves three steps: surrounding prey, hunting prey, and attacking prey.

#### 2.2.1. Surrounding Prey

The surrounding behavior of grey wolves is mathematically represented by Equations (1) and (2):

(1) $$\vec{D}=\left|\vec{V}.\vec{P_{p}}\left(t\right)-\vec{P}(t)\right|$$

(2) $$\vec{P}\left(t+1\right)=\vec{P_{p}}\left(t\right)-\vec{U}.\vec{D}$$

where ‘t’ denotes the round number, $\vec{U}$ and $\vec{V}$ are vectors, $\vec{P_{p}}$ denotes the location of the prey, $\vec{P}$ is the location of the wolf, and $\vec{D}$ denotes the gap between the wolf and prey. The coefficient vectors $\vec{U}$ and $\vec{V}$ are computed using Equation (3):

(3) $$\vec{U}=2\;\vec{a}\;.\vec{r_{1}}-\vec{a}$$

(4) $$\vec{V}=2\;.\vec{r_{2}}$$

where $\vec{r_{1}}$ and $\vec{r_{2}}$ are vectors with values in a range of [0, 1], and the value of $\vec{a}$ increases linearly from 0 to 2 throughout the iterations. The grey wolf updates its position by modifying $\vec{U}$ and $\vec{V}$ along with random vectors $\vec{r_{1}}$ and $\vec{r_{2}}$ and which aid the wolf in approaching the prey.

#### 2.2.2. Hunting Prey

The hunting strategy of the GWO is orchestrated by optimal solutions provided by the alpha, beta, and delta wolves, enabling the prediction of prey locations. The locations of omega wolves are dynamically modified according to the coordinates of the alpha, beta, and delta wolves. The following equation can be used to represent this process:

(5) $${\vec{Y}}_{\alpha }=\left|{\vec{N}}_{1}.{\vec{P}}_{\alpha }-\vec{P}\right|,{\vec{Y}}_{\beta }=\left|{\vec{N}}_{2}.{\vec{P}}_{\beta }-\vec{P}\right|,{\vec{Y}}_{\delta }=\left|{\vec{N}}_{3}.{\vec{P}}_{\delta }-\vec{P}\right|$$

where $\vec{P}$ denotes the wolf’s current position; ${\vec{Y}}_{\alpha }$, ${\vec{Y}}_{\beta }$, and ${\vec{Y}}_{\delta }$ denote the current locations of alpha, beta, and delta wolves, respectively; ${\vec{D}}_{\alpha }$, ${\vec{D}}_{\beta },$ and ${\vec{D}}_{\delta }$ represent the updated locations of alpha, beta, and delta wolves, respectively; and ${\vec{N}}_{1}$, ${\vec{N}}_{2},$ and ${\vec{N}}_{3}$ are coefficient vectors. The absolute positions of wolves ${\vec{P}}_{1}$, ${\vec{P}}_{2},$ and ${\vec{P}}_{3}$ are calculated using the following equations:

(6) $${\vec{P}}_{1}={\vec{P}}_{\alpha }-{\vec{R}}_{1}.\left({\vec{Y}}_{\alpha }\right),{\vec{P}}_{2}={\vec{P}}_{\beta }-{\vec{R}}_{2}.\left({\vec{Y}}_{\beta }\right),{\vec{P}}_{3}={\vec{P}}_{\delta }-{\vec{R}}_{3}.\left({\vec{Y}}_{\delta }\right)$$

(7) $$\vec{P}\left(t+1\right)=\frac{{\vec{P}}_{1}+{\vec{P}}_{2}+{\vec{P}}_{3}}{3}$$

where ${\vec{R}}_{1}$, ${\vec{R}}_{2},$ and ${\vec{R}}_{3}$ represent random vectors, and *t* specifies the current iteration.

#### 2.2.3. Attacking Prey

To execute an attack, the parameter $\vec{a}$ is reduced and the values of vector $\vec{U}$ are concurrently decreased by *a* within the interval of [−2*a*, 2*a*]. The parameter *a* gradually diminished from 2 to 0 in successive iterations. If the random values of $\vec{U}$ fall within the interval of [−1, 1], the subsequent location of the searching agent will be the point lying between its present position and the location of the prey. If |U| < 1, the wolf is compelled to initiate an attack on the prey. Conversely, if |U| > 1, the wolves separate from each other in their pursuit of locating the prey. In the proposed algorithm, cluster heads are represented by grey wolves, with the base station serving as the prey.

## 3. Proposed Method

**Figure 1:**
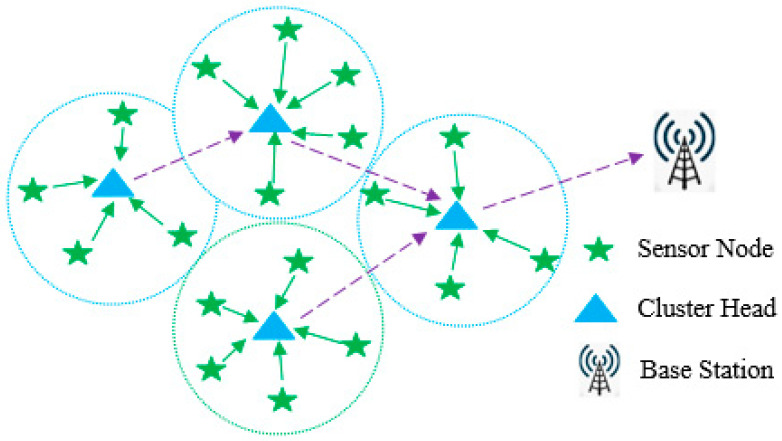
Cluster-based WSN.

The hierarchical architecture of a WSN is depicted in Figure 1. Sensor nodes are arranged into discrete clusters, where the head of each cluster gathers information from all member nodes. The information collected by the cluster head is transmitted to the base station in a multi-hop manner. This type of hierarchical architecture is known as the clustering architecture of a WSN. The proposed method used two algorithms for routing and clustering, respectively. GWO is used to identify the best route to reach the base station from each cluster head, and a chaotic genetic algorithm is employed for clustering. Because the sensor nodes operate on batteries, it is crucial to optimize both energy usage and sensor lifetime when designing the clustering and routing processes.

### 3.1. Clustering Using Chaotic Genetic Algorithm

**Figure 2:**
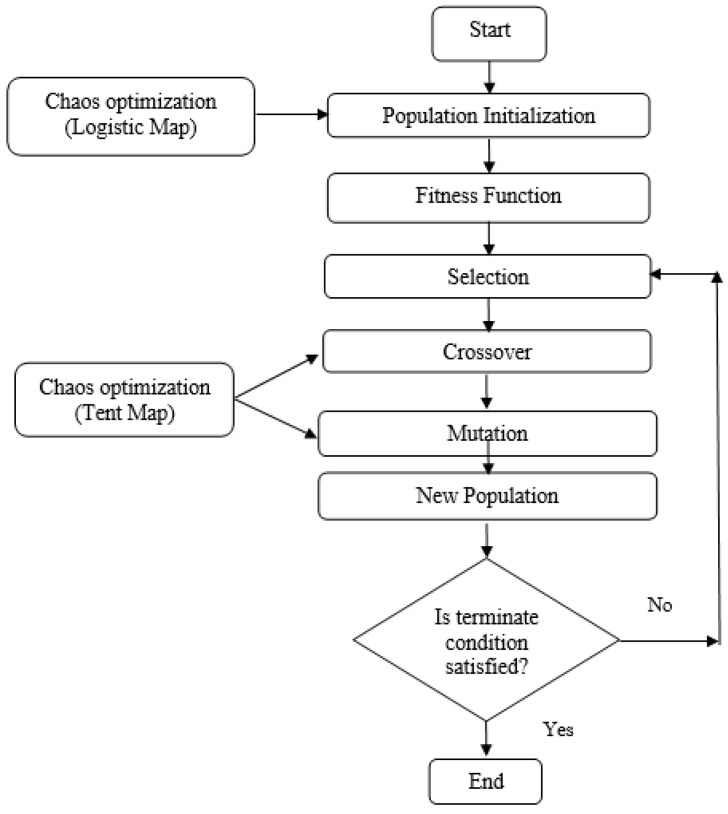
Chaotic genetic algorithm.

The proposed method generates clusters using various phases of a genetic algorithm. Chaotic dynamics are used in the initial population, crossover, and mutation phases of the algorithm. The initial population process uses chaotic systems, such as logistic maps, whereas the crossover and mutation processes use tent maps. Figure 2 illustrates the operation of the chaotic genetic algorithm. First, solutions are randomly initialized to generate an initial population, with each solution composed of one or more chromosomes consisting of a string or collection of characters. After the initial population is generated, higher-performing individuals are selected using the fitness function. Next, a crossover operation is performed on two individuals at random such that their parents can create two new individuals as offspring.

A mutation procedure is performed to create a new generation, with the fitness levels of the parents and new children evaluated using the fitness function. Parents are formed through selection, and the next generation is created through mutation. Towards the end, the algorithm mixes the new population with half of the individuals who perform well. If the new population does not satisfy the termination criterion, the algorithm is passed to the next generation; otherwise, it terminates. The objective of embedding chaotic features into a genetic algorithm is to improve search capabilities and introduce randomness into the optimization process.

#### 3.1.1. Chromosome Representation

**Figure 3:**
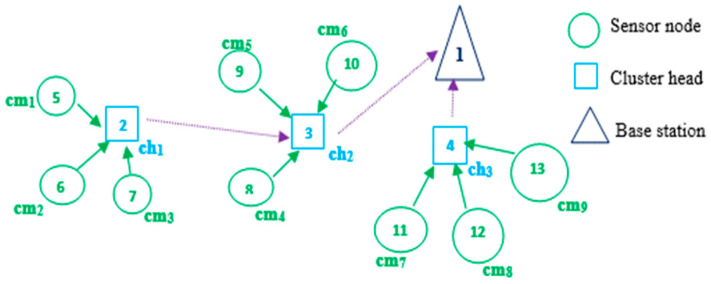
WSN clustering.

**Figure 4:**
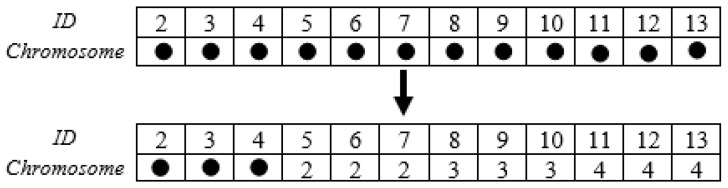
Real-number coding of chromosome for cluster head selection.

With sensor nodes represented as genes, chromosomes are used to represent a random selection of nodes from the network. Basic sensor nodes like Mica2/MicaZ motes were utilized in this study. These nodes are known for their low power consumption and straightforward sensing capabilities. They are typically deployed in large quantities to provide extensive coverage across an area. As a solution to identifying suitable cluster heads, real-number coding is used to generate chromosomes. Each chromosome has a length of *n* − 1, where *n* represents the total number of sensors as well as the base station. Each node in the network is identified using a real number, with the base station identified as 1 (ID(sink) = 1), each cluster head node identified as ID(ch_i_) = I + 1, and each cluster member node identified as ID(cm_i_) = i + 1 + n_CH_. The size of each cluster head is indicated by n_CH_. For example, in the WSN depicted in Figure 3, the identities of cluster heads 2, 3, and 4 are 1, 2, and 3, respectively, while that of member node 6 is 2. Figure 4 shows the process of chromosome coding for cluster head selection in the network depicted in Figure 3. The network has 14 nodes, with nodes 2, 3, and 4 being cluster heads and the remaining nodes being cluster members.

#### 3.1.2. Population Initialization

There are *q* randomly produced chromosomes in the initial population P_init_, where each chromosome corresponds to a potential solution. The initial population is denoted as

(8) $$P_{init}=\left\{{chr}_{m1},{chr}_{m2},\ldots \ldots ,{chr}_{mi},\ldots \ldots {,chr}_{mn}\right\}\;\mathrm{and}\;{chr}_{mi}\epsilon [a_{mi},b_{mi}]$$

where *n* represents the dimension of variables chr_mi_, m = 1, 2, 3, ……… N, and i = 1, 2, 3 ……… *n*.

The local convergence and high tolerance of a genetic algorithm result from randomness. Hence, the rule of randomness given by chaotic dynamics replaces the random process in the population initialization. Chaos offers pseudo-randomness and ergodicity characteristics in a random process, thereby avoiding the premature convergence of results. Several chaotic maps—including the Chebyshev, Gauss, logistic, piecewise, singer, and tent maps—are used to generate the initial population. The tent map is used during the crossover and mutation processes, whereas the logistic map is used to create the initial population. As expressed by Equation (9), the logistic map yields the chaotic variables, where *k* represents the iteration number and r represents the control parameter.

(9) $$x_{i}^{k+1}=rx_{i}^{k}(1-x_{i}^{k})\;\mathrm{where}\;x_{i}^{0}\epsilon [0,1]\;\mathrm{and}\;i=1,2\ldots \ldots n,\;k=0,1,2\ldots \ldots .$$

The value of r lies within [0, 4], where r = 4 corresponds to a completely chaotic system. After the value of r is determined, the chaotic-type initial value is determined using Equation (10):

(10) $$x_{mi}^{0}=({chr}_{mi}-a_{mi})/(b_{mi}-a_{mi})$$

The chaotic variables $x_{i}^{0},x_{i}^{1},x_{i}^{2},\ldots \ldots \ldots ,x_{i}^{k}$ are generated according to Equation (9) and subsequently mapped from the chaos space to the solution space using Equation (11).

(11) $${chr}_{mi}=a_{mi}+(b_{mi}-a_{mi})x_{mi}^{k}\;\mathrm{where}\;m=1,2,\ldots N\;\mathrm{and}\;i=1,2\ldots \ldots \ldots n.$$

Thus, the initial population is created based on the locations of the n values in the chaotic environment following the aforementioned calculations.

#### 3.1.3. Fitness Function

Chromosome quality in a genetic algorithm is assessed using a fitness function. Because the cluster heads send gathered information to the base station via several hops, the maximum energy remaining in each cluster head node corresponds to chromosome fitness. As the energy depletion of cluster heads during periods of high load determines the network’s energy efficiency, reducing the energy consumption of the cluster heads is crucial for extending the network lifetime. The remaining energy of the nodes is estimated using a first-order radio (FOR) model. Among a set of cluster head candidates, choose the one which satisfies the criterion. Through this way of selecting cluster heads, the load is balanced among the nodes in the network. The fitness value of each chromosome (${Chr}_{i}$) is given by

(12) $$fit({Chr}_{i})=E_{res}(i)$$

The i^th^ node’s residual energy ($E_{res}(i)$) is calculated using Equation (13):

(13) $$E_{res}(i)=E_{init}(i)-E_{cons}(i)$$

where $E_{init}(i)$ is the initial energy of i^th^ node, and $E_{cons}(i)$ represents the energy consumed by the nodes. The energy consumption ($E_{cons}$(i)) of i^th^ node is calculated using Equation (14):

(14) $$E_{cons}(i)=E_{rx}(i)-E_{tx}(i)$$

where $E_{rx}(i)$ and $E_{tx}\left(i\right)$ are the reception and transmission energies of i^th^ node respectively. The reception energy ($E_{rx}(i)$) of i^th^ node is calculated using Equation (15):

(15) $$E_{rx}(i)=pl\times E_{elec}(i)$$

where pl is the length of the packet to be received and $E_{elec}(i)$ is the electrical energy consumption of i^th^ node. The transmission energy of the nodes is calculated using Equation (16) for the free space model and Equation (17) for the multispace model.

(16) $$E_{tx}(i)=pl\times E_{elec}(i)+pl\times \varepsilon_{fs}\times d^{2},d<d_{0}$$

(17) $$E_{tx}(i)=pl\times E_{elec}(i)+pl\times \varepsilon_{mp}\times d^{4},d>d_{0}$$

where $d_{0}=\frac{\varepsilon_{fs}}{\varepsilon_{mp}}$. Here d is the distance between the nodes, $\varepsilon_{fs}$ is the free space energy and, $\varepsilon_{mp}$ is the multispace energy.

Thus, chromosomes that consume lower amounts of energy or have maximum residual energy are selected as cluster heads. Chromosomes with a high residual energy are associated with higher fitness values; hence, the fitness function is directly proportional to the residual energy of each node, as shown in Equation (18). Therefore, the solution with maximum fitness value after the given number of iterations is considered as the best solution.

(18) $$fit(i)\propto E_{res}(i)$$

After selecting the cluster heads, they broadcast a message called CH_DEC to all other nodes in the network. This message contains the ID and location of the sender cluster head within the deployment area. Upon receiving this broadcast, every node in the network prepares a candidate list based on the information provided. This list includes the ID of each cluster head and a corresponding cost value. The cost value is determined by factors such as the distance between the node and the cluster head, the maximum transmission range of the network, and the initial and residual energy levels of the node. It can be defined as

(19) $$c(i)={(E}_{res}(i)/E_{init}(i))+(D_{ij}/D_{max})$$

where D_ij_ is the distance between i^th^ node to j^th^ cluster head and D_max_ is the maximum transmission range. The nodes join with cluster head based on the cost value. Cluster heads are selected based on having the lowest cost value, which is determined by factors like distance, network range, and energy levels. Additionally, a balancing factor is employed to control the cluster size, considering the total number of nodes and the number of selected cluster heads. Nodes then send a request message, JOIN_MESG, to the identified cluster head. Upon receiving this request, the cluster head evaluates whether it can include the node in its cluster. If it can, it responds with a JOIN_ACN message. Otherwise, it sends a JOIN_RJ message. If a node receives a JOIN_RJ message, it should seek another cluster head with the lowest cost value from the candidate list. This process continues until the node successfully joins a cluster. This way, clusters are formed based on the calculated costs.

#### 3.1.4. Selection

Chromosomes with the highest fitness values are selected as parent chromosomes for the crossover and mutation operators. The proposed algorithm selects high-quality chromosomes to pass on from elite individuals directly to the next generation using elitist selection. The elitist selection approach improves convergence speed over the roulette wheel selection method by directly reproducing elite individuals in each successive generation.

#### 3.1.5. Crossover and Mutation

The tent-map chaotic system is used for crossover and mutation operations. Chaotic behavior can influence the mixing of genetic material in a manner that is less predictable than traditional crossover methods. Here, the two child chromosomes ${x_{c1}=(x}_{c1}^{0},x_{c1}^{1},x_{c1}^{2},\ldots \ldots \ldots ,x_{c1}^{k}$) and ${x_{c2}=(x}_{c2}^{0},x_{c2}^{1},x_{c2}^{2},\ldots \ldots \ldots ,x_{c2}^{k}$) are generated from a pair of parent chromosomes ${x_{p1}=(x}_{p1}^{0},x_{p1}^{1},x_{p1}^{2},\ldots \ldots \ldots ,x_{p1}^{k})$ and ${x_{p2}=(x}_{p2}^{0},x_{p2}^{1},x_{p2}^{2},\ldots \ldots \ldots ,x_{p2}^{k})$ as follows:

(20) $$x_{c1}^{i}=\frac{1}{2}[(1-\alpha )x_{p1}^{i}+(1+\alpha )x_{p2}^{i}]$$

(21) $$x_{c2}^{i}=\frac{1}{2}[(1+\alpha )x_{p1}^{i}+(1-\alpha )x_{p2}^{i}]$$

where α is generated as

(22) $$\alpha =\left\{\begin{array}{c} {\left(2u\right)}^{\frac{1}{\left(\eta_{c}+1\right)}},\;\;\mathrm{if}\;u\leq 0.5\\ {\left(\frac{1}{2\left(1-u\right)}\right)}^{\frac{1}{\left(\eta_{c}+1\right)}}\;\mathrm{otherwise} \end{array}\right.$$

Here, u is a random integer between 0 and 1. The distribution index of the crossover operator is denoted by $\eta_{c}$. The value of u is generated using the tent map, with the k^th^ iteration of u calculated as follows:

(23) $$u^{k+1}=\left\{\begin{array}{c} ru^{k}\;\;\;\mathrm{if}\;u^{k}<\frac{1}{2}\\ r\left(1-u^{k}\right)\;\;\mathrm{if}\;u^{k}>\frac{1}{2}\; \end{array}\right.\;\mathrm{where}\;u^{0}\epsilon [0,1]\;\mathrm{and}\;k=0,1,2\ldots \ldots .$$

where r is the control parameter satisfying 1<r<2, thereby ensuring chaotic behavior. For solution $x_{i}$, the mutation operation is expressed as follows:

(24) $$x_{i}^{*}=x_{i}+(x_{i}^{u}-x_{i}^{l})\delta_{i}$$

where $x_{i}^{u}$ and $x_{i}^{l}$ are the upper and lower bounds of $x_{i}$, respectively, and

(25) $$\delta_{i}=\left\{\begin{array}{c} {\left(2u_{i}\right)}^{\frac{1}{\left(\eta_{m}+1\right)}}\;\;\mathrm{if}\;\;u_{i}<0.5\\ {1-(2\left(1-u_{i}\right)))}^{\frac{1}{\left(\eta_{m}+1\right)}}\;\;\mathrm{otherwise} \end{array}\right.$$

where ‘$u_{i}$’ is a random integer between 0 and 1. The distribution index of the mutation operator is denoted by $\eta_{m}$. The value of ‘$u_{i}$’ is generated using tent map and the k^th^ iteration of u_i_ is calculated as follows.

(26) $$u_{i}^{k+1}=\left\{\begin{array}{c} ru_{i}^{k}\;\;\;\mathrm{if}\;u_{i}^{k}<\frac{1}{2}\\ r\left(1-u_{i}^{k}\right)\;\;\mathrm{if}\;u_{i}^{k}>\frac{1}{2}\; \end{array}\right.\;\mathrm{where}\;u_{i}^{0}\epsilon [0,1]\;\mathrm{and}\;i=1,2\ldots \ldots n,\;k=0,1,2\ldots \ldots .$$

where ‘r’ is the control parameter and 1<r<2, the system exhibits chaotic behavior. The generated child chromosome is finalized as a cluster head for the given network. Thus, cluster heads are selected using a chaotic genetic algorithm that considers the residual energy of the nodes during each network round.

### 3.2. Routing Using Grey Wolf Optimization

In GWO, alpha, beta, delta, and omega wolves represent different levels of the family structure, mimicking the social environment and hunting tactics of real-world grey wolves. These wolves work together to optimize the fitness environment with the goal of determining the most suitable path for a particular problem. There are three phases of routing using GWO:

#### 3.2.1. Initialization of Wolves

A population of grey wolves is initialized, where each wolf corresponds to a routing path for the problem and the positions of wolves on the routing path represent potential solutions. The solution must establish a route to reach the base station through the network’s subsequent cluster heads. Because the wolves’ positions are randomly generated upon initialization, a random number is assigned to each cluster head. Letting $P_{i}=\{{CH}_{1},{CH}_{2},\ldots \ldots \ldots \ldots {CH}_{n})$ be the i^th^ solution in the population, ${CH}_{i}$ represents i^th^ cluster head and n denotes the number of clusters. The population of grey wolves CH_i_ (i = 1, 2, …, n) is randomly initiated and each cluster head is initialized with a random number CH_i_ = rand(0,1). CH_j_, which represents the next succeeding cluster head of CH_i_ in the routing path to reach the base station, is determined according to the following equation:

(27) $${CH}_{j}=Choose(Neighbors\left({CH}_{i}\right),k)$$

where the Choose function returns the *k^th^* cluster head from the neighbors of *CH_i_*_,_ and the Neighbors function determines the potential neighboring cluster heads. The value of k is determined as

(28) $$k={CH}_{i}\times |Neighbors\left({CH}_{i}\right)|$$

#### 3.2.2. Routing Fitness Evaluation

The energy usage, length, and hop count of the routing path are key parameters that determine the fitness value for routing. Based on these considerations, a fitness function was formulated to determine the most efficient route for arriving at the base station.

The energy usage ${EP}_{i}$ of the path from the cluster head ${CH}_{i}$ to the base station is calculated as follows:

(29) $${EP}_{i}=\sum_{i=1}^{m}\frac{1}{E_{nexthop({CH}_{i})}}$$

where $E_{nexthop({CH}_{i})}$ is the residual energy of the subsequent hop from the cluster head ${CH}_{i}$.

Equation (30) defines the route length required to reach the base station ${DP}_{i}$ from the cluster head ${CH}_{i}$ as follows:

(30) $${DP}_{i}=\sum_{i=1}^{m}d({CH}_{i},nexthop({CH}_{i}))$$

where $nexthop\left({CH}_{i}\right)$ denotes the cluster head identified as the next succeeding hop for ${CH}_{i}$. The distance between ${CH}_{i}$ and ${CH}_{j}$ is represented by the function $d\left({CH}_{i},{CH}_{j}\right)$.

Because additional cluster head hops in a given path result in higher energy consumption, the total number of hops between the base station HP_i_ and cluster head CH_i_ is defined as follows:

(31) $${HP}_{i}=\sum_{i=1}^{m}nexthopCount({CH}_{i})$$

where nexthopCount(CH_i_) denotes the hop length between the base station and cluster head.

The selection of a routing path considers variables including path length, number of hops, and energy consumption. Specifically, the routing path with the highest fitness value is associated with a shorter total distance, fewer hops, and lower energy consumption per hop. Thus, the routing fitness value RFV is evaluated as

(32) RFV=(α×EP)+(β×DP)+(γ×HP)

where α, β, and γ are weighing factors satisfying α + β + γ = 1.

#### 3.2.3. Wolf Position Updating

The positions of beta, delta, and alpha wolves are updated according to Equations (5)–(7), representing the first-, second-, and third-best solutions, respectively. These updated positions are determined by averaging the current locations of the wolves. To standardize each wolf’s most recent position within the interval [0, 1], values falling outside that interval are adjusted as follows:

- If $pos\left({CH}_{i}\right)\leq 0$ then $pos\left({CH}_{i}\right)=\max\left(r1,r2,r3\right)$;
- If $pos({CH}_{i})\geq 1$ then $pos\left({CH}_{i}\right)=1$.

## 4. Simulation and Results

The proposed chaotic genetic algorithm was evaluated using a MATLAB simulation under the assertion that the chaotic dynamics used in the algorithm are sufficient to achieve energy-efficient clustering. However, evaluating the efficacy of GWO-based routing involves assessing key parameters—including network lifetime, energy efficiency, throughput, and routing overhead—using a network simulation tool (Network Simulator 2.35). Although MATLAB is used for simulating the clustering process, the performance graphs were created using NS2.35. In NS2.35, various network scenarios are generated and obtain trace files containing crucial simulation events data, such as packet transmissions and receptions, among other metrics. These trace files were then formatted appropriately to ensure compatibility with Xgraph, a visualization tool within NS2.35. Xgraph offers a wide array of options for customizing plot appearance, including line styles, colors, and axis labels. Moreover, it allows for the plotting of multiple datasets on the same graph, facilitating easy comparison between different scenarios. After generating the plots, the visualized data are meticulously analyzed to derive meaningful insights into the performance of network simulation. 

Table 1 presents the simulation configuration and parameters, along with their respective values or methodologies. The 100 × 100 m^2^ network area size offers a realistic yet computationally manageable environment for sensor network simulation, accommodating diverse topologies. With 50–250 nodes, scalability across networks of varying sizes can be explored, from smaller setups where efficiency is less critical to larger ones. Placing the base station at (90,90) simplifies evaluation by centralizing data aggregation and transmission efficiency. A 2 m transmission range ensures ample connectivity without excessive interference or energy use. Initial node energy at 3 J provides a realistic starting point. Transmission and receiving energies (0.6 J and 0.2 J) are chosen based on theoretical estimates. A packet length of 4000 bits aligns with typical sensor network data sizes. A population size of 20 and 100 generations balances computational efficiency with thorough solution exploration. The elitist selection method maintains the best-performing solutions, aiding convergence towards optimal outcomes. 

The efficacy of the proposed CGA-GWO algorithm was evaluated through a comparative experiment with existing schemes, including the low-energy adaptive clustering hierarchy genetic algorithm (LEACH-GA) [21], fuzzy GWO [19], and genetic-algorithm-based unequal clustering and routing (GA-UCR) [20]. Performance metrics including the active node count, remaining network energy, packet transmission ratio, clustering overhead, and routing overhead were measured to evaluate each method’s efficacy. The performance measures are explained as follows:

Number of alive nodes: This metric measures the number of nodes in the network whose reserved energy has not yet depleted.
Average remaining energy: This metric indicates the average amount of energy still available among sensor nodes active in the network.
Percentage of packets received: This metric represents the ratio of packets successfully received by a node to the total number of packets it attempted to send within the network.
Clustering overhead: This metric refers to the additional control and communication costs associated with the establishment and maintenance of clusters within the network.
Routing overhead: This metric refers to the additional communication and computational costs incurred by routing protocols in managing and optimizing data transmission within the network.

The classic LEACH protocol presents several shortcomings that render it less suitable for comparison with the proposed CGA-GWO algorithm. These drawbacks include unequal cluster sizes, inefficiency with network size, limited support for multi-hop communication, and static network assumptions. Due to these inherent limitations, this research work does not include LEACH classic in the comparative analysis. Furthermore, according to the literature, LEACH-GA exhibits significant enhancements over classic LEACH in critical performance areas such as network lifetime, energy efficiency, throughput, and routing overhead. Moreover, existing research [20,27,28] indicates that alternative algorithms such as LEACH-GA, Fuzzy-GWO, and GA-UCR offer notable improvements over LEACH classic in key performance metrics such as network lifetime, energy efficiency, throughput, and routing overhead. These algorithms have demonstrated enhanced effectiveness in managing energy consumption, extending network lifetime, optimizing data throughput, and reducing routing overhead when compared to the traditional LEACH approach.

**Figure 5:**
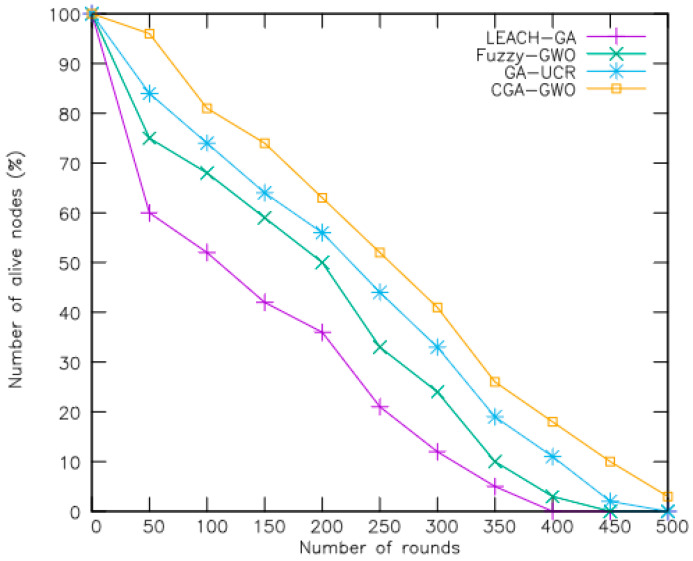
Number of live nodes in each network with respect to number of rounds.

Figure 5 depicts a plot of the number of live nodes in a 200-node network with respect to the number of rounds. According to the simulation results, the CGA-GWO algorithm exhibited more live nodes, surpassing GA-UCR, fuzzy GWO, and LEACH-GA by 15%, 30%, and 50%, respectively. In LEACH-GA, nodes are designated as cluster heads based on an optimized probability, leading to increased energy consumption. Moreover, the dead node count increased when the base station moved beyond the sensor field. In contrast, fuzzy GWO selects cluster heads according to residual energy, specifically targeting nodes with values that surpass the average remaining energy of all cluster heads. This involves the calculation of a fitness function that considers increased energy consumption, resulting in a higher number of dead nodes within the network. In GA-UCR, a genetic is applied for both cluster head selection and routing, combining the two schemes into a single chromosome. Consequently, energy consumption is a parameter in the fitness function calculation. As compared to other related algorithms, CGA-GWO shows a higher number of alive nodes as the number of iterations increases, as illustrated in Figure 5. This improvement is attributed to the integration of chaotic features in the genetic algorithm and the strategic selection of nodes as cluster heads. CGA-GWO enhances search efficiency and increases the number of alive nodes by integrating chaotic elements into its genetic algorithm framework. 

**Figure 6:**
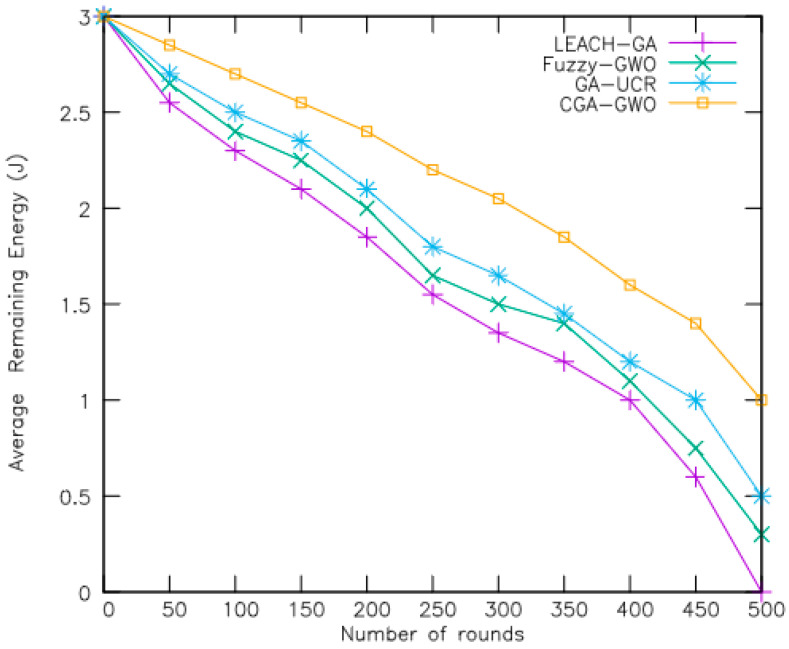
Average remaining energy with respect to number of rounds.

Figure 6 presents a plot of average remaining energy in a 200-node network over an increasing number of rounds. Here, CGA-GWO showed approximately 17%, 22%, and 30% more residual energy than GA-UCR, fuzzy-GWO, and LEACH-GA, respectively, by selecting nodes with the highest fitness values as cluster heads. The network can sustain operation for up to 700 rounds when the average remaining energy of the proposed algorithm CGA-GWO reaches 1 J by the 500th round. Specifically, the proposed algorithm determines suitable nodes for every generation of the population for energy-efficient routing. In addition, CGA-GWO selects chromosomes that consume less energy—i.e., have the maximum residual energy. These chromosomes are associated with the highest fitness values, establishing a direct proportionality between the fitness function and residual energy. Thus, the selection of cluster heads is facilitated by a chaotic genetic algorithm that relies on the residual energy of nodes in the network. This approach enhances the efficiency of cluster head selection and contributes to the overall energy optimization in the network.

**Figure 7:**
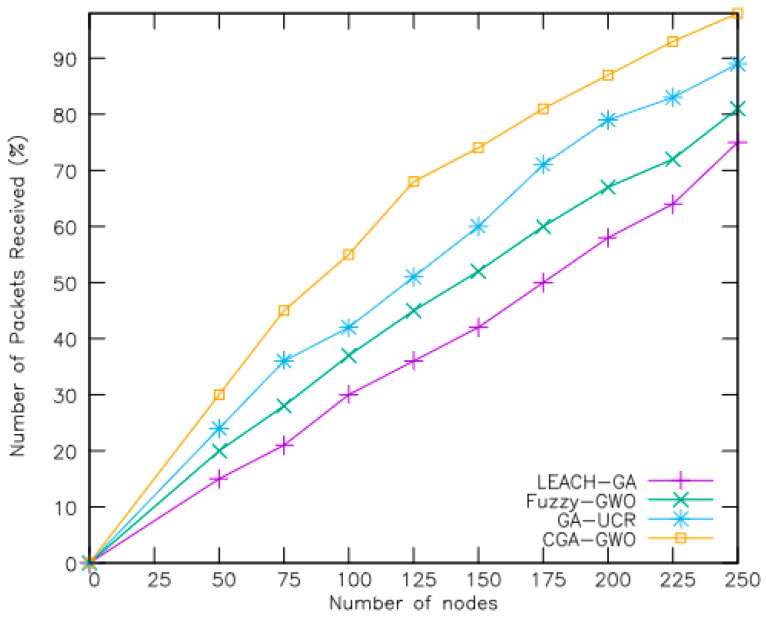
Number of packets received by increasing nodes.

Figure 7 shows the percentage of packets received by considering the total number of nodes in the network. For each algorithm, the percentage of packets received increased along with the number of nodes. Nonetheless, CGA-GWO outperformed LEACH-GA, fuzzy-GWO, and GA-UCR by 38%, 27%, and 14%, respectively, in terms of the percentage of packets received. The proposed CGA-GWO algorithm achieves a packet reception rate of 63%, which surpasses that of other related schemes. These results can be attributed to the fact that the GA-UCR algorithm neglects the existence of energy holes or hotspots formed during the routing process. In contrast, CGA-GWO maintains an increased number of delivered packets owing to its fitness function, which effectively minimizes packet loss during data transmission. To further reduce packet loss, CGA-GWO constructs an efficient path that does not contain dead nodes. Conversely, LEACH-GA and fuzzy GWO have high packet drop ratios stemming from inappropriate cluster head selection.

**Figure 8:**
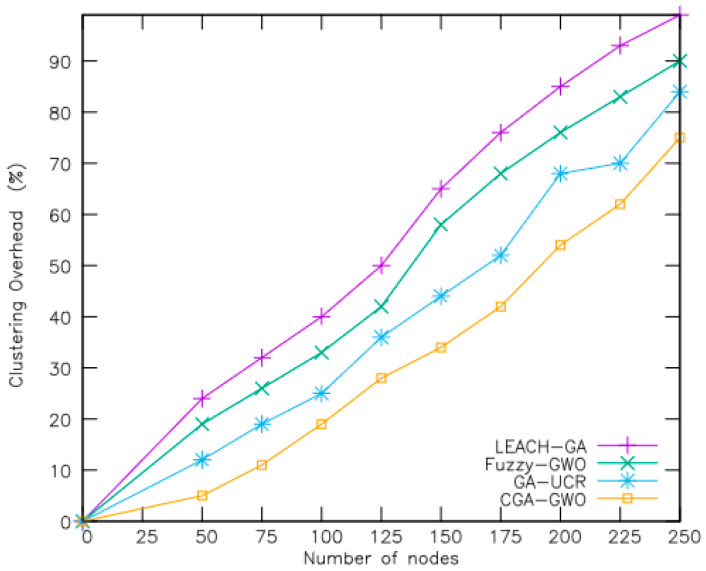
Clustering overhead.

In Figure 8, the clustering overhead of the CGA-GWO is compared with that of the three baselines by varying the number of nodes. CGA-GWO exhibited overhead reductions of 41%, 34%, and 11% compared with LEACH-GA, Fuzzy-GWO, and GA-UCR, respectively, as the proposed clustering fitness function selects high-residual energy nodes as cluster heads. Furthermore, the incorporation of chaotic features reduces the involvement of heavily loaded sensor nodes in the clustering process. In addition, the CGA-GWO algorithm employs an elitist selection method to select high-quality chromosomes, ensuring the transmission of elite individuals to subsequent generations. This ensures that higher-quality genes participate in each successive generation’s clustering process. In contrast, fuzzy GWO incurs a higher clustering overhead owing to repeated transmissions, whereas GA-UCR uses an intercluster separation fitness function to select cluster heads, resulting in increased overhead owing to the sizes of clusters. Ultimately, CGA-GWO employs a chaotic genetic algorithm to identify suitable cluster heads, minimizing the overhead associated with the clustering process.

**Figure 9:**
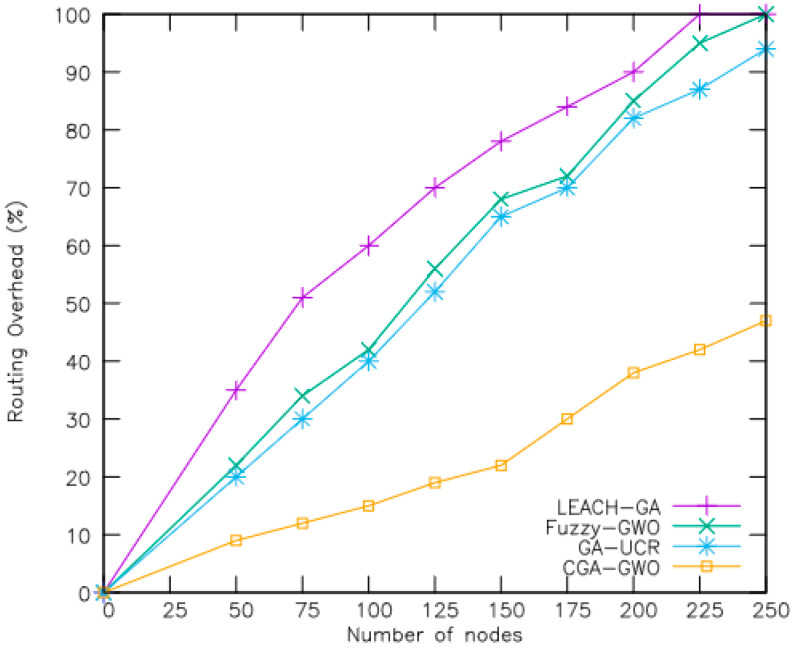
Routing overhead.

In Figure 9, the routing overheads of the different schemes are plotted for varying numbers of nodes, with CGA-GWO exhibiting a 47% reduction in overhead compared with the baselines. This is attributed to the consideration of factors such as the number of hops per path and residual energy per hop when selecting a routing path. By considering these factors, the fitness function facilitates the identification of an efficient route to the base station. The optimization aims to reduce the hop count along the path, resulting in a more efficient routing path compared with that determined by the baselines. In addition, the wolves’ arrangement and directional movements play crucial roles in determining the optimal routing solution. In GA-UCR, the classic genetic algorithm is employed to navigate data toward the base station given the NP-hard nature of the problem. In fuzzy GWO, nodes along the routing path remove the corresponding packets from their queues, leading to increased packet retransmissions and, consequently, a higher routing overhead.

The fitness function is specifically designed to incorporate these factors, aiding in the identification of efficient routes to the base station. This optimization strategy aims to minimize the hop count along the path, resulting in a superior routing path compared to other algorithms. Additionally, the arrangement of wolves and their directional movement within CGA-GWO’s genetic algorithm framework play a crucial role in determining the optimal solution for routing within the population. In contrast, GA-UCR utilizes a classical genetic algorithm to navigate data toward the base station, acknowledging the NP-Hard nature of the problem. Meanwhile, Fuzzy-GWO experiences increased routing overhead due to nodes along the routing path removing corresponding packets from their queues, leading to heightened packet retransmissions. In summary, CGA-GWO’s comprehensive approach to routing optimization, considering factors such as hop count and residual energy, coupled with its efficient genetic algorithm framework, contributes to its significant reduction in routing overhead compared to other schemes.

## 5. Conclusions

In this study, a chaotic genetic algorithm was combined with the GWO technique to obtain an energy-efficient solution for cluster-based optimal routing. The innovation of the proposed algorithm lies in the utilization of chaotic dynamics to select high-quality chromosomes for the clustering process and determine the routing path, with the GWO mechanism implemented to minimize packet retransmission. In addition, the residual energy levels of the nodes in each network round are considered using the chaotic genetic algorithm during the selection of cluster heads. Furthermore, the optimal routing solution is determined by the wolves’ positions and trajectories. Extensive simulation experiments were conducted to assess the functionality of the proposed CGA-GWO algorithm using both MATLAB and NS2 simulators. The simulation outcomes indicate that CGA-GWO enhances network performance across a wide range of metrics.

## Figures and Tables

**Table 1:** Parameter settings for simulation.

Parameter/Scheme	Value/Method
Network area size	100 × 100 m^2^
Number of nodes	50–250
Location of base station	(90, 90)
Transmission range	2 m
Initial energy of each node	3 J
Transmission energy	0.6 J
Receiving energy	0.2 J
$E_{elec}$	50 nJ/bit
$\varepsilon_{fs}$	15 pJ/bit/m^2^
$\varepsilon_{mp}$	0.0015 pJ/bit/m^4^
Packet length (*pl*)	4000 bits
Population size	20
Number of generations	100
Selection method	Elitist selection method

## Data Availability

The original contributions presented in the study are included in the article, further inquiries can be directed to the corresponding authors.
